# Validation of a new predictive model to improve risk stratification in bronchopulmonary dysplasia

**DOI:** 10.1038/s41598-019-56355-5

**Published:** 2020-01-17

**Authors:** Gustavo Nino, Awais Mansoor, Geovanny F. Perez, Maria Arroyo, Xilei Xuchen, Jered Weinstock, Kyle Salka, Mariam Said, Ranniery Acuña-Cordero, Monica P. Sossa-Briceño, Carlos E. Rodríguez-Martínez, Marius Linguraru

**Affiliations:** 1 Division of Pediatric Pulmonary and Sleep Medicine. Children’s National Medical Center, George Washington University, Washington, DC USA; 2 Sheikh Zayed Institute for Pediatric Surgical Innovation, Children’s National Medical Center, Washington, DC USA; 3 Division of Neonatology. Children’s National Medical Center, George Washington University, Washington, DC USA; 4 Department of Pediatrics, George Washington University, Washington, DC USA; 5 Department of Radiology, George Washington University, Washington, DC USA; 6 Department of Biomedical Engineering, George Washington University, Washington, DC USA; 7 Department of Pediatric Pulmonology, Hospital Militar Central, Department of Pediatrics, School of Medicine, Universidad Militar Nueva Granada, Bogotá, Colombia; 8 Department of Internal Medicine, School of Medicine, Universidad Nacional de Colombia, Bogotá, Colombia; 9 Department of Pediatrics, School of Medicine, Universidad Nacional de Colombia, Bogotá, Colombia; 10 Department of Pediatric Pulmonology and Pediatric Critical Care Medicine, School of Medicine, Universidad El Bosque, Bogota, Colombia

**Keywords:** Health care, Medical research

## Abstract

We need a better risk stratification system for the increasing number of survivors of extreme prematurity suffering the most severe forms of bronchopulmonary dysplasia (BPD). However, there is still a paucity of studies providing scientific evidence to guide future updates of BPD severity definitions. Our goal was to validate a new predictive model for BPD severity that incorporates respiratory assessments beyond 36 weeks postmenstrual age (PMA). We hypothesized that this approach improves BPD risk assessment, particularly in extremely premature infants. This is a longitudinal cohort of premature infants (≤32 weeks PMA, n = 188; Washington D.C). We performed receiver operating characteristic analysis to define optimal BPD severity levels using the duration of supplementary O2 as predictor and respiratory hospitalization after discharge as outcome. Internal validation included lung X-ray imaging and phenotypical characterization of BPD severity levels. External validation was conducted in an independent longitudinal cohort of premature infants (≤36 weeks PMA, n = 130; Bogota). We found that incorporating the total number of days requiring O2 (without restricting at 36 weeks PMA) improved the prediction of respiratory outcomes according to BPD severity. In addition, we defined a new severity category (level IV) with prolonged exposure to supplemental O2 (≥120 days) that has the highest risk of respiratory hospitalizations after discharge. We confirmed these findings in our validation cohort using ambulatory determination of O2 requirements. In conclusion, a new predictive model for BPD severity that incorporates respiratory assessments beyond 36 weeks improves risk stratification and should be considered when updating current BPD severity definitions.

## Introduction

Bronchopulmonary dysplasia (BPD) is a serious respiratory condition of premature newborn infants that is characterized by abnormal development of the lungs and pulmonary vasculature^1–4^. Despite the variability in diagnostic criteria and clinical practice, the requirement for supplemental O2 at 28 days has been used as the BPD definition for more than three decades^5–11^. Given that the need for supplemental O2 at 36 weeks postmenstrual age (PMA) predicts subsequent respiratory outcomes^9^, a BPD severity classification was initially constructed with three levels (mild, moderate, severe) using 28 days and 36 weeks PMA as evaluation time points^5,6^. These BPD severity levels have passed the test of time demonstrating adequate correlation with structural lung changes^12–16^, and respiratory morbidity after NICU discharge^11^. However, the progress in NICU care has led to an increasing number of babies surviving extreme prematurity^17^, which is posing a challenge for the quantification of BPD severity because they often need longer NICU hospitalizations and require supplemental O2 beyond 36 weeks PMA. Current BPD definitions cannot capture this type of information because the most severe BPD category (level III) only includes the fraction of inspired O2 (FiO2) and/or the need of mechanical support and/or positive pressure at 36 weeks PMA^5,6^.

The central premise of this study is that a new predictive model that incorporates respiratory assessments beyond 36 weeks PMA can provide critical information about BPD severity and the risk for subsequent respiratory morbidity. Specifically, we hypothesized that including the total number of days requiring O2 (without restricting at 36 weeks PMA) in BPD severity definitions will: 1) improve the prediction of respiratory outcomes according to BPD severity, and 2) define a new severity category (level IV) that encompasses babies born extremely premature with prolonged O2 exposure. To test these hypotheses we used a data-driven approach to construct a new BPD severity classification in four steps: 1) determination of the optimal discrimination thresholds of the days of O2 requirement to predict respiratory hospitalization after NICU discharge (primary classifier of BPD severity) using receiver operating characteristic (ROC) analysis in a cohort of premature infants (n = 188; Washington D.C. discovery cohort); 2) validation of the new clinical BPD severity scale using lung X-ray scoring as independent secondary marker of BPD severity in the same study cohort; 3) characterization of the resultant clusters of BPD severity using individual clinical features and formal statistical testing between groups; and 4) testing the new predictive risk model for premature infants that incorporates the total number of O2 dependence days beyond 36 weeks PMA in an independent cohort with longitudinal data covering 12 months after NICU discharge (n = 130; Bogota, Colombia, validation cohort).

We feel our current study will have a high impact in the field providing timely new information to accelerate ongoing efforts to update BPD severity definitions based on scientific evidence^6,18,19^. In addition, we are defining new BPD subsets of severity, which may be crucial to investigate disease mechanisms and to evaluate future interventions in clinical trials.

## Methods

### Study population

The primary cohort was comprised of 188 premature infants (≤32 weeks PMA) admitted to the NICU at Children’s National Health System (CNHS) in Washington, D.C. The validation cohort included 130 premature infants (≤36 weeks PMA) admitted to the NICU at The Hospital Militar Central and the Hospital Universitario Clinica San Rafael in Bogota, Colombia. All individuals enrolled had at least 12 months of longitudinal data after NICU discharge. Baseline characteristics of all study subjects are presented in Table 1. Study design of these cohorts is further detailed in Online Repository. All research was performed in accordance with relevant guidelines/regulations approved by the Institutional Review Board (IRB) of CNHS in Washington D.C. and by the local ethics board in the Hospital Militar Central and the Hospital Universitario Clinica San Rafael in the Bogota cohort. The IRB of CNHS in Washington D.C. approved the study and granted a waiver of informed consent given that this research involved materials (medical records) collected solely for clinical indications. Informed consent was obtained from legal guardians in all participants in the Bogota cohort.

**Table 1:** Characteristics of study cohorts.

Clinical variables	Washington Cohort (n = 188)	Bogota Cohort (n = 130)	P value
Gestational age at birth (weeks, mean, SD)	27.02 (3.04)	30.65 (2.76)	<0.001
<28 weeks % (n)	55.3 (104)	13.8 (18)	<0.001
28–31 weeks% (n)	34.6 (65)	42.3 (55)	0.164
≥32 weeks % (n)	10.1 (19)	43.8 (57)	<0.001
Male gender % (n)	59 (111)	59 (77)	0.973
Birth weight (grams, mean, SD)	996 (452)	1438 (418)	<0.001
Maternal smoking % (n)	0.05 (10)	0.02 (2)	0.054
Duration of oxygen therapy (days, mean, SD)	74 (49.8)	108 (78.4)	<0.001
≤89 days % (n)	60 (114)	45 (59)	0.007
90–119 days % (n)	20.7 (39)	18.5 (24)	0.613
120 or more days % (n)	18.6 (35)	36.1 (47)	0.001
Duration of mechanical ventilation (days, mean, SD)	25.1 (26.3)	9.8 (13.3)	<0.001
Respiratory Hospitalizations (mean/yr, SD)	0.79 (1.3)	0.83 (1.1)	0.746
Proportion of subjects with respiratory hospitalization % (n)	43.6 (82)	49.2 (64)	0.323

### Definition of BPD and respiratory outcomes

BPD was defined according to NHLBI workshop^5,6^. “No BPD” was defined as <28 days of supplemental O2. Mild BPD included infants who received O2 or respiratory support for >28 days but were on room air at 36 weeks PMA. In this study O2 days were defined as a day in which the infant received any amount supplemental O2 even if it was intermittent. In all cases the indication of O2 supplementation was hypoxemia and the target was >90% SpO2. Infants with moderate BPD required supplemental oxygen, <30% FiO2, at 36 weeks PMA. Finally, severe BPD was classified as the use of >30% FiO2 and/or the need of mechanical support and/or positive pressure at 36 weeks PMA. Premature infants receiving nasal airflow ≥3 LPM at 36 weeks PMA were also included in the severe BPD category^6^. Our main outcome was the binary presence of ≥1 respiratory hospitalization in the first year of life. We only counted a respiratory hospitalization as those in which the primary complaint was any type of respiratory sign or symptom (e.g. cough, nasal/chest congestion, wheezing, respiratory distress, hypoxemia, etc).

### Lung imaging analysis

All individuals enrolled in our primary cohort had a baseline chest radiograph (CXR) performed near discharge (between 36–41 weeks PMA), which was used for lung disease quantification. The visual scoring was conducted blindly and independently by two pediatric pulmonologists (GN and GP) using a modified grading system described by Greenough *et al*.^12–14^. The scoring system had three signatures of BPD: 1) fibrosis/interstitial opacities; 2) cystic/coarse elements; and 3) hyperinflation. Each image was assigned a blinded total score(0–8) based on a global grading system for each of these lung disease parameters. Computer-assisted image intensity standardization and automatic segmentation (quadrants)^20–22^, are further described in supplementary methods.

### Statistical analysis

Descriptive statistics are presented as mean and standard deviation (SD). Group comparisons were performed using t-test, Mann-Whitney U-test and chi^2^-test, as appropriate. Binary logistic regression models were used to evaluate the predictive value of the different variables, using respiratory hospitalization as the primary outcome. This binary outcome was also used to construct receiver operating characteristic (ROC) curves and estimate the area under the curve (AUC) for each one. The calculations were made with STATA version 14 (StataCorp. Stata Statistical Software: Release 14. College Station, TX. 2015).

## Results

### Construction of a BPD severity scoring system in a cohort of premature infants

**Figure 1:**
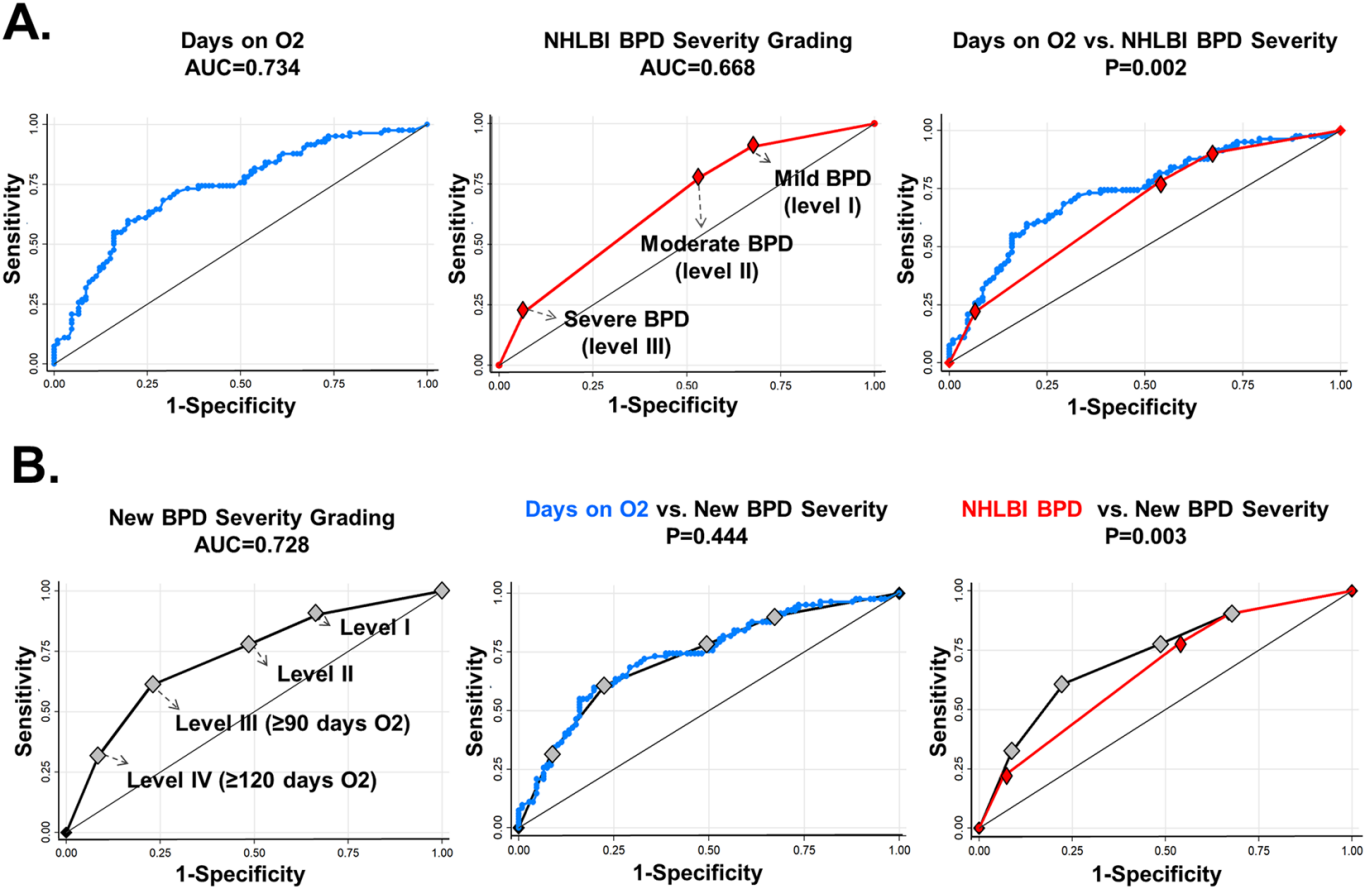
Construction of a data-driven BPD severity scoring system. Using respiratory hospitalization as binary outcome, we found that (**A**) the total days on O2 is a better predictor than NHLBI BPD severity due to the lack of accuracy beyond the data point assessment at 36 weeks PMA (level II). (**B**) New BPD levels adding ≥90 days (level III) and ≥120 days (Level IV) provide equivalent prediction to the total days on O2 and are superior to current NHLBI BPD severity definitions.

**Figure 2:**
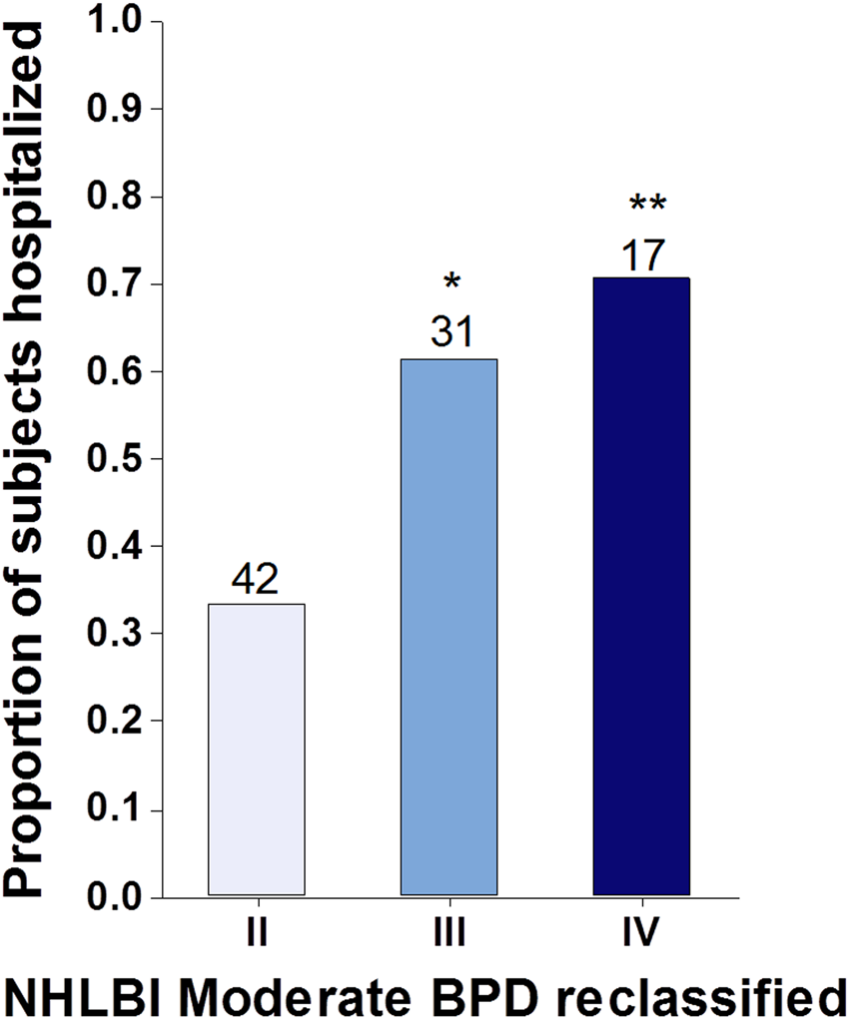
New BPD severity scoring system improves risk prediction. Individuals initially classified as moderate BPD according to NHLBI definitions (level II) were reclassified as level III and level IV based on O2 requirements beyond 36 weeks PMA. Reclassified individuals had higher probability of respiratory hospitalization. *p < 0.05, **p < 0.01 relative to new level II.

We first examined the area under the receiver operating characteristic (ROC) curves (AUC) of different predictor(s) of the binary presence of ≥1 respiratory hospitalization in the first year of life in a cohort of premature infants (n = 188; Washington, D.C.) (Table 2). We found that the total number of days requiring supplemental O2 was the best predictor for our primary BPD severity classifier (AUC = 0.734). This predictor was better than the current BPD severity grading (AUC = 0.66) due to the lack of accuracy beyond the data point assessment at 36 weeks PMA (level II, Fig. 1A). We next calculated the sensitivity, specificity, positive predictive value, and negative predictive value for each data point of the best predictor (number of days on O2) to define discrimination threshold points after 36 weeks PMA (Table S1). Figure 1B shows that optimal fitting of the AUC can be obtained from the current BPD severity classification adding ≥90 days on O2 as a new criteria for severe BPD (level III) and generating a new severity level defined as O2 requirements ≥120 days (level IV). Specifically, we found that ≥90 days had >70% accuracy predicting respiratory hospitalizations after discharge and that ≥120 days had >90% specificity to predict the same outcome (Table S1). Using these new criteria we were able to optimize the current BPD severity classification improving the overall accuracy of the grading system. We found that this new BPD severity scale (4 levels) had equivalent performance to the total number of days on supplemental O2 (Fig. 1B) and was superior to the current BPD severity classification (3 levels) predicting respiratory hospitalization in premature infants (Fig. 1B). In addition, we identified that 53% of the individuals initially classified as moderate BPD (level II) based on current definition (<0.3 FiO2 needs without PAP at 36 weeks PMA independently of days on O2) were in fact either level III (≥90 days on O2) or level IV (≥120 days on O2) using the new criteria (Fig. 2). Importantly, these misclassified individuals had a significantly higher probability of respiratory hospitalizations (Fig. 2), confirming that the assessment at 36 weeks PMA alone is inadequate to stratify BPD risk. Collectively, these data provided construct and criterion validation for our new respiratory disease severity system and demonstrated its superiority against current BPD definitions predicting respiratory outcomes.

**Table 2:** Predictors of respiratory hospitalization in the primary cohort.

Clinical predictor	ROC AUC*	95% Conf. Interval	
Days requiring supplemental O2	0.7348	0.66076	0.80885
Days requiring mechanical ventilation	0.6797	0.59895	0.76037
NHLBI BPD severity grading	0.6767	0.60235	0.75108
Birth weight	0.6731	0.59193	0.75435
Gestational age	0.6448	0.56288	0.72663
Apgar 1 min	0.5851	0.50111	0.66909
Apgar 5 min	0.5825	0.49894	0.66599
Cesarean section	0.5681	0.49677	0.63934
Chorioamnionitis	0.5129	0.47406	0.55176
Maternal smoking	0.5107	0.47931	0.54212
Gender	0.504	0.43092	0.5771
**Examination at NICU discharge**			
Supplemental O2 at discharge	0.6189	0.55045	0.68742
Heart rate	0.5474	0.46151	0.63324
Respiratory rate	0.5314	0.4461	0.61669
Wheezing	0.5036	0.48521	0.52206

*Receiver operating characteristic (ROC) area under the curve (AUC).

### Content validity of the new respiratory disease severity system using independent lung imaging grading

**Figure 3:**
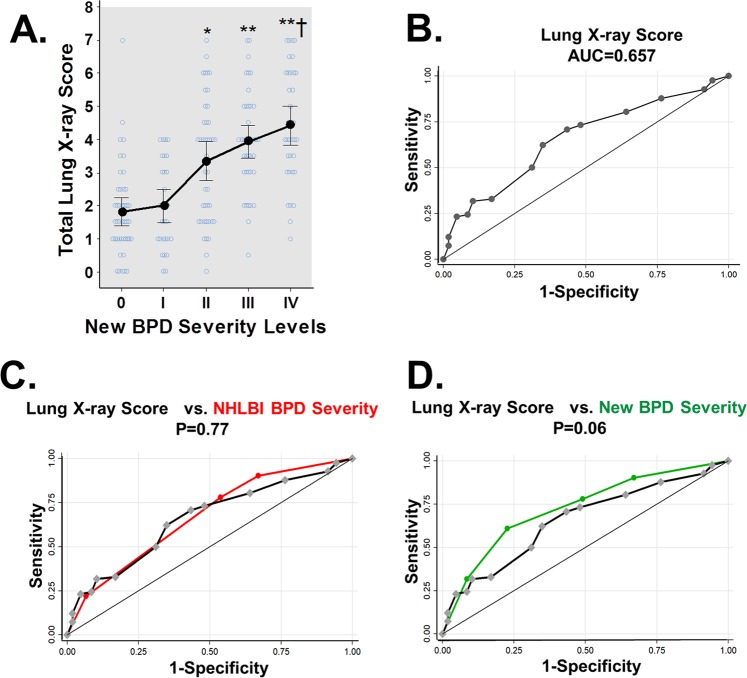
Validation of new BPD severity levels using lung X-ray imaging. (**A**) Total CXR scores correlated with new BPD levels and (**B**) predicted respiratory hospitalization with (**C**) equivalent performance than NHLBI BPD severity classification. (**D**) New BPD severity levels trended to be superior CXR scores. *p < 0.05 for level I vs. II, **p < 0.01 for level I vs. III and IV, ^†^p < 0.05 for level II vs. I

The risk of respiratory hospitalization in premature babies is only one criteria of BPD severity and may not be directly related to the severity of lung disease. Thus, we next validated our scoring system using lung X-ray scoring as an independent secondary marker of BPD severity in the same study. We found that our scoring system strongly correlated with an increasing trend of BPD severity in lung X-ray across the 4 new levels (Fig. 3A). ROC analysis showed that total CXR severity score had equivalent performance to the current BPD severity classification predicting ≥1 respiratory hospitalization in the first year of life (Fig. 3B,C). The performance of the proposed BPD severity system (4 levels) trended to be superior to CXR scoring alone (BPD score 0.724 vs. CXR score 0.682; p = 0.06, Fig. 3D).

### Defining new BPD severity clusters using individual clinical features

**Figure 4:**
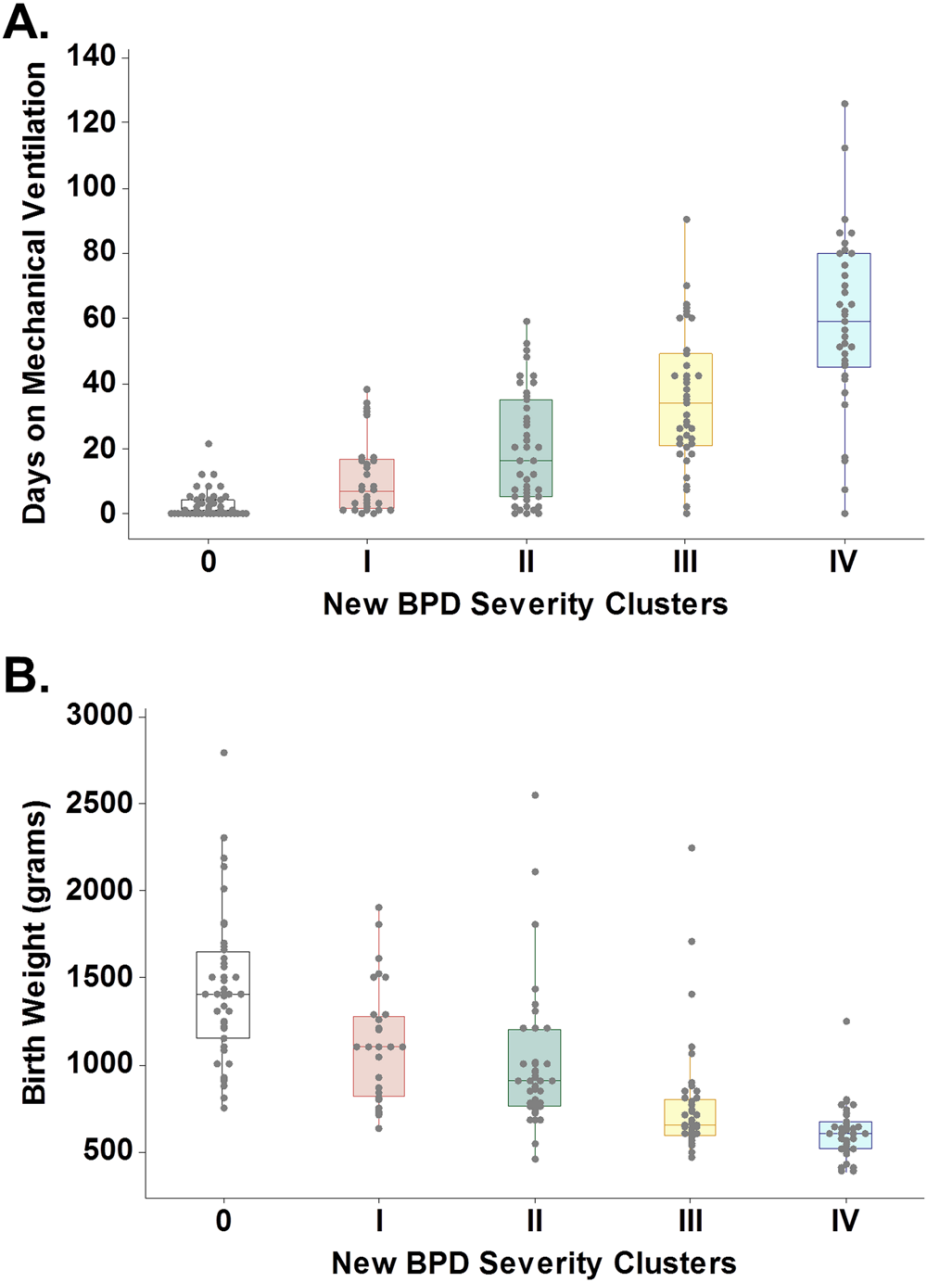
Characterization of new BPD severity clusters. Box plots showing that (**A**) total days of mechanical ventilation and (**B**) birth weight were significantly different across the 4 new BPD severity groups (ANOVA p < 0.0001).

To further validate the new BPD severity grading (4 levels), we examined whether the proposed categories truly represent different clusters of BPD severity in which the individuals of each group have different characteristics. Given that birth weight and mechanical ventilation have been recently identified as BPD clustering factors^23^, we tested the hypothesis that these variables also discriminate our BPD severity groups. As shown in Fig. 4, birth weight and mechanical ventilation were significantly different across the new 4 severity levels of BPD in our study cohort. Additional individual clinical features of each severity group are presented in Table 3. Comparison of the first two levels (BPD level I and II) showed similar risk of subsequent respiratory admissions, however, individuals with moderate BPD had worse lung X-ray scores and more days on mechanical ventilation. Individuals in the new level III (severe BPD) had lower GA, lower birth weight, and significantly higher risk of subsequent respiratory admissions (OR = 7 relative to non-BPD group). The new BPD severity level IV (premature babies with ≥120 days of supplemental O2) included individuals with prolonged mechanical ventilatory support (mean 59 days, 95% CI 53.1–64.9 days) and extreme prematurity (lower birth weight and lower PMA). Notably, premature babies in the new severity level IV had the worst CXR scores and the highest risk for subsequent hospitalization after discharge (OR = 12.6 relative to non-BPD group). Furthermore, these babies had significantly higher risk of having pediatric intensive care unit (PICU) admission (OR = 9.4 relative to non-BPD group). These data further validated the proposed severity grading system as it identifies different clusters of BPD severity, including a new level IV of “very severe BPD” that encompasses a group of vulnerable infants with exceedingly high risk for severe respiratory complications after NICU discharge.

**Table 3:** Characteristics of BPD severity clusters in the primary cohort.

Clinical variables	Non-BPD (n = 43)	BPD Level I (n = 29)	BPD Level II (n = 42)	BPD Level III (n = 39)	BPD Level IV (n = 35)	P value*
Days requiring O2 (mean, 95% CI)	9.6 (5.5, 13.5)	41 (36.1, 45.8)	73.2 (69.1, 77.2)	105.8 (101.6, 110)	145.9 (141.5, 150.4)	<0.001
Days mechanical ventilation (mean, 95% CI)	2.7 (−2.6, 8.1)	11.3 (4.8, 17.8)	19.8 (14.4, 25.2)	35.2 (29.6, 40.8)	59.0 (53.1, 64.9)	<0.001
Birth weight (grams, mean, 95% CI)	1430 (1324, 1535)	1116 (987, 1244)	1006 (899, 1113)	767 (656, 878)	609 (492, 726)	<0.001
Gestational age (weeks, mean, 95% CI)	30.4 (29.8, 31.0)	28.2 (27.4, 28.9)	26.8 (26.2, 27.4)	25.4 (24.8, 26.0)	23.9 (23.2, 24.6)	<0.001
Apgar score 1 min (mean, 95% CI)	6.5 (5.8, 7.1)	5.4 (4.5, 6.2)	4.3 (3.6, 5.0)	4.2 (3.5, 4.9)	3.2 (2.5, 3.9)	<0.001
Apgar score 5 min (mean, 95% CI)	8.1 (7.6, 8.7)	7.5 (6.8, 8.2)	6.4 (5.9, 7.0)	6.8 (6.2, 7.4)	5.7 (5.1, 6.3)	<0.001
Male gender % (n)	53.5 (23)	55.2 (16)	61.9 (26)	64.1(25)	60 (21)	0.862
**Lung X-ray**						
Total CXR score (0–8, 95% CI)	1.8 (1.3, 2.3)	2 (1.4, 2.6)	3.3 (2.9, 3.8)	3.9 (3.4, 4.4)	4.4 (3.9, 5.0)	<0.001
Fibrosis/interstitial opacities score (0–4, 95% CI)	0.9 (0.6, 1.3)	0.9 (0.5, 1.4)	2 (1.7, 2.4)	2.6 (2.2, 2.9)	2.8 (2.4, 3.2)	<0.001
Cystic/coarse elements score (0–2, 95% CI)	0.05 (−0.1, 0.2)	0.1 (−0.1, 0.3)	0.29 (0.1, 0.4)	0.36 (0.2, 0.5)	0.49 (0.3, 0.6)	<0.001
Hyperinflation score (0–2, 95% CI)	0.85 (0.69, 1.01)	0.97 (0.77, 1.16)	1.06 (0.90, 1.22)	1.04 (0.87, 1.21)	1.16 (0.98, 1.34)	0.133
**Respiratory outcomes**						
Respiratory hospitalizations (mean/yr, 95% CI)	0.21 (−0.1, 0.6)	0.72 (0.3, 1.2)	0.67 (0.3, 1.0)	0.95 (0.6, 1.3)	1.51 (1.1, 1.9)	<0.001
Proportion of subjects with respiratory hospitalization % (n)	18.6 (8)	34.4 (10)	33.3 (14)	61.5 (24)	74.2 (26)	<0.001
Risk of respiratory hospitalization (OR to non-BPD)	—	2.3 (0.8, 6.8)	2.2 (0.8, 6.0)	7 (2.6, 19.1)	12.6 (4.3, 37.2)	<0.001
PICU admissions (mean/yr, 95% CI)	0.05 (−0.1, 0.2)	0.07 (−0.1, 0.3)	0.17 (0.0, 0.3)	0.23 (0.1, 0.4)	0.46 (0.3, 0.6)	0.004
Proportion of subjects with PICU admission % (n)	4.7 (2)	6.9 (2)	16.7 (7)	17.9 (7)	31.4 (11)	<0.001
Risk of PICU admission (OR to non-BPD)	—	1.5 (0.2, 11.4)	4.1 (0.8, 21.0)	4.5 (0.9, 23.1)	9.4 (1.9, 46.0)	0.001

*p values obtained by ANOVA for continuous variables and logistic regression for binary variables.

### Testing a new predictive risk model for premature infants that incorporates the total number of O2 dependence beyond the 36 weeks PMA

A potential limitation of using ≥90 days or ≥120 days of supplemental O2 to identify high-risk infants with BPD is that they may be discharged from the hospital before these times can be actually estimated. Notably, in our primary cohort (n = 188; Washington, D.C.), we were able to estimate these time points prior to discharge in all subjects classified as BPD level III or level IV; thus this is likely applicable to other institutions. Nonetheless, Washington, D.C is a large referral center for severe prematurity with 58% of the infants included in our study staying in the NICU beyond 36 weeks PMA. Thus, we used an independent cohort (n = 130; Bogota, Colombia) to examine the risk of respiratory hospitalizations in premature infants requiring supplemental O2 beyond 36 weeks PMA under different conditions. Differences between our study cohorts are presented in Table 1. Our validation cohort was located in a low middle income tropical country, included preterm infants ≤36 weeks PMA and there were no survivors of <25 weeks PMA. Given Bogota’s high altitude (8660 ft. above sea level), premature individuals were prone to have hypoxemia and required longer days on supplemental O2 overall (Table 1). The cohort was set as part of a Kangaroo Mother Care (KMC) program that encouraged early home discharges so that babies requiring supplemental O2 beyond 36 weeks PMA had the final determination of the total days of supplemental O2 in the ambulatory setting. This was done objectively during outpatient visits testing the physiological need for O2 (room air challenge) as part of the KMC program. The binary presence of ≥1 respiratory hospitalization in the first year of life was the primary outcome, and it was defined identically as in our validation cohort.

**Figure 5:**
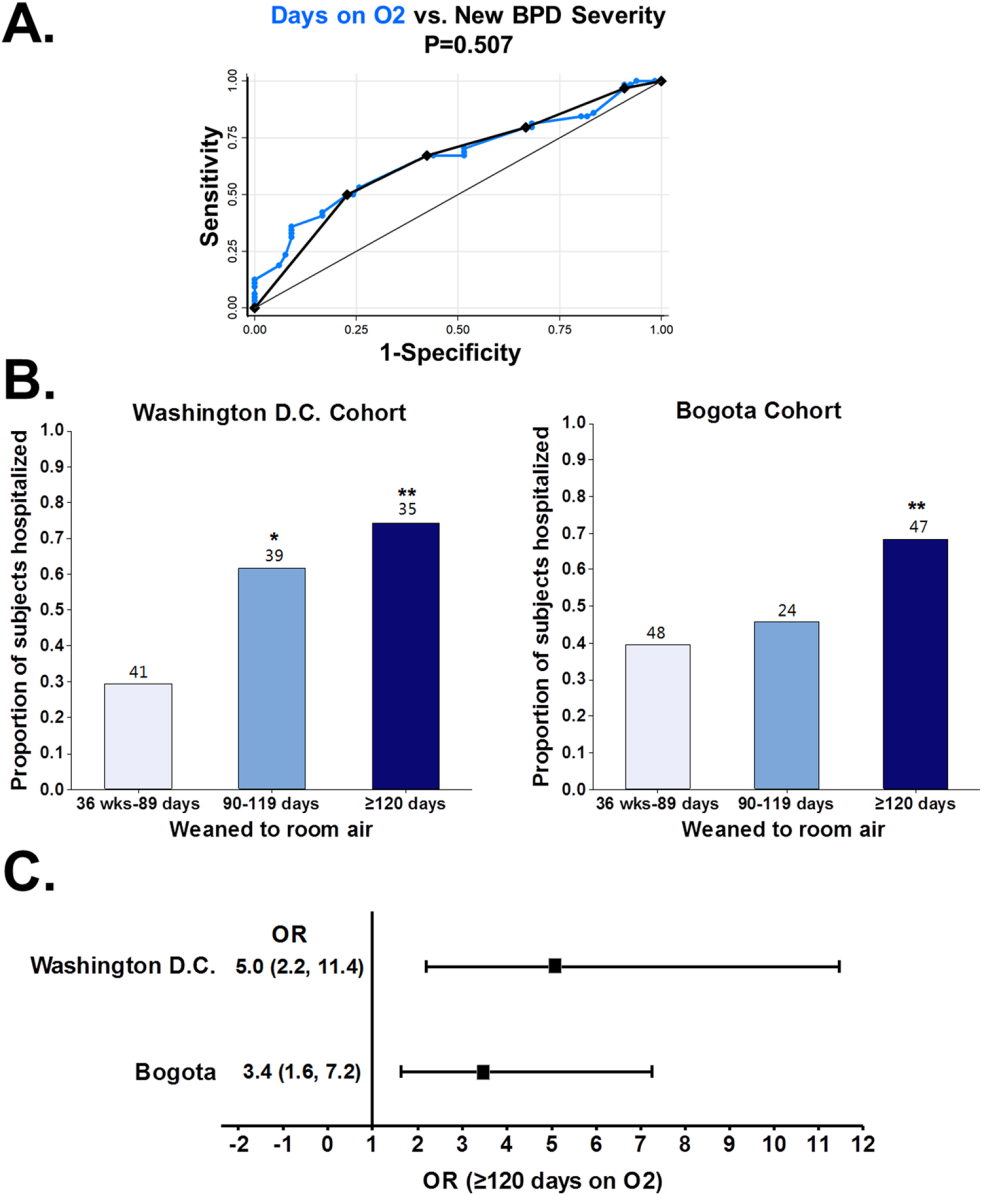
External validation of a new predictive BPD risk model that incorporates the assessments beyond the 36 weeks PMA. (**A**) In a validation cohort (Bogota, n = 130), the total days on O2 and the new BPD severity levels predicted respiratory hospitalization. In both cohorts, (**B**) the probability of respiratory hospitalization increased with longer time to be weaned to room air, and (**C**) the highest risk was observed in individuals with ≥120 days on O2. Data presented as odds ratio (OR) and 95% confidence intervals.

We found that the total number of days requiring supplemental O2 was also a significant predictor of respiratory hospitalization in the Bogota cohort (AUC 0.66, Fig. 5A). In agreement with our findings in the Washington, D.C. cohort, the same 4 levels proposed for BPD severity classification fitted the AUC and provided almost identical risk prediction (AUC 0.65, Fig. 5A). We also observed an increasing trend in the probability of respiratory hospitalization with a longer time to be weaned to room air after 36 weeks PMA (Fig. 5B). The highest probability of respiratory hospitalization in both cohorts was seen in premature babies that were still on O2 by 120 days PMA (Washington, D.C. = 0.74 and Bogota = 0.68). Lastly, our predictive model (logistic regression), demonstrated that premature babies requiring supplemental O2 ≥120 days had significantly increased risk of respiratory hospitalization in both the Washington, D.C. cohort (OR = 5.0) and in the Bogota cohort (OR = 3.4) (Fig. 5C). Collectively, these results demonstrated the utility of the proposed BPD severity system in two distinct clinical scenarios and confirmed that respiratory evaluations beyond 36 weeks PMA provide critical information to predict outcomes and thus should be considered when updating current BPD severity definitions.

## Discussion

The main finding of this study is that the respiratory assessment beyond 36 weeks PMA provides critical information about BPD severity improving the risk assessment for future respiratory morbidity. Specifically, in our primary cohort (premature babies ≤32 weeks PMA, Washington, DC, n = 188), we found that incorporating the total number of days requiring O2 (without restricting at 36 PMA) improved the prediction of respiratory outcomes according to BPD severity. In addition, we were able to define a new severity category (level IV) of premature babies with prolonged exposure to supplemental O2 (≥120 days) who had the highest risk of respiratory hospitalizations after NICU discharge. We validated our new grading system using quantification of lung X-rays as an independent marker of BPD severity. We also were able to characterize new severity clusters using individual clinical features in the same study cohort. Furthermore, we validated the new predictive risk model of BPD that incorporates the total number of days requiring O2 beyond 36 weeks PMA in an independent cohort of premature infants (Bogota cohort, n = 130) that included preterm infants (≤36 weeks PMA). In our validation cohort, we demonstrated that ambulatory determination of O2 requirements beyond 36 weeks PMA shows similar results to those obtained during inpatient evaluation (Fig. 5), indicating that BPD severity is a dynamic process that may need to continue after NICU discharge. Taken together, our data provides compelling evidence that points of evaluation beyond 36 weeks PMA should be considered when updating current BPD severity definitions^23^.

It is becoming increasingly clear that current BPD severity definitions need to be updated^6,18,19^. Clinical practice has changed dramatically and our knowledge about the pathobiology of this condition has improved considerably. Moreover, the progress in NICU care has led to an increasing number of babies surviving extreme prematurity^17^ making the assessment at 36 weeks PMA insufficient to evaluate BPD severity because these babies often require supplemental O2 beyond that time point. It is also clear that we need better BPD risk stratification to predict long term outcomes because nearly half of the infants born ≤29 weeks PMA are re-hospitalized in the first year of life^24^. In addition, developing an accurate BPD severity grading system is critically needed to improve our understanding of the most severe forms of BPD and to enable the identification of novel mechanisms and biomarkers of respiratory disease progression in prematurity. The latter would be a major milestone to develop new therapeutic strategies and to improve the design of future clinical trials.

Notably, despite the high-impact of this area of research, there is still a paucity of studies providing scientific evidence to guide future updates of BPD severity definitions. Our current study contributes significantly to filling this gap presenting a novel data-driven approach to optimize BPD severity grading and risk prediction. For this purpose, we used receiver operating characteristic (ROC) curves, a robust method to define classes (usually severity levels in medical conditions) based on the accuracy (sensitivity and specificity) across different discrimination thresholds^25,26^. As the initial binary BPD severity outcome, we used ≥1 respiratory hospitalization in the first year of life. We chose this dichotomous parameter because it is a hard variable (easily defined and quantified) that is clinically relevant for BPD severity and risk assessment. We subsequently used disease quantification in lung X-ray to validate our findings obtained through our primary BPD binary classifier. Importantly, using ROC curves, we identified that the best predictor was the total number of days requiring supplemental O2. This is in agreement with prior studies^9^, and underlies why the number of days requiring supplemental O2 is used in the current BPD severity definitions, in which 36 weeks PMA is the last point of assessment^5,6^.

Interestingly, we identified that the number of days requiring supplemental O2 continues discriminating individuals with a higher probability of future respiratory hospitalizations after 36 weeks PMA. Beyond this time point, we found increasing specificity and improved accuracy with an inflection point around 90 days PMA (>70% accuracy and >75% specificity) and a plateau near 120 days PMA (≈92% specificity). In other words, we found that evaluation at 36 weeks PMA alone was insufficient to accurately estimate the risk of future of hospitalizations; however assessments after 120 days did not significantly improve the prediction of this outcome. Although there are procedures to calculate the precise class thresholds in ROC curves^26,27^, we decided to simply adapt current BPD severity classifications to facilitate implementation. We added ≥90 days (~3 months) of O2 supplementation to the current severe BPD category (level III) and generated a new level IV defined as ≥120 days (~4 months) of O2 requirements. Current BPD severity definitions only recommend assessments at 36 weeks PMA^5,6^ without including late data points (i.e. 120 days) to identify the severest BPD cases. Indeed, our new grading system identified that many individuals (53%) initially classified as “moderate” in fact had prolonged exposure to O2 (≥90 days or ≥120 days) that conferred a much higher risk of hospitalization after discharge (Fig. 2). We also found that within the initial category of severe BPD (level III), there is a subset of premature infants with a significantly more severe BPD phenotype characterized by prolonged exposure to O2 (≥120 days), lower birth weight, and more severe disease in lung-X rays. These premature infants also had the highest risk of respiratory hospitalizations after NICU discharge with a significantly higher probability of requiring PICU admission. A limitation of our study is that our cohorts did not include premature infants on mechanical support beyond NICU discharge; however, these babies would likely be included in this new category given that this group was characterized by prolonged mechanical ventilatory support (mean 59 days). Given their exceedingly high risk for severe complications, we believe these vulnerable infants with “very severe BPD” (new level IV) may require a detailed assessment of their upper and large airways^28^, lung parenchyma structure^12–16^, pulmonary vasculature^29^, and ventilatory responses^30,31^, as well as intense surveillance by a specialized multi-disciplinary team during and after NICU discharge^32^. We feel that babies with ≥120 days of O2 requirements should be considered in a different severity category because we found they have a much greater risk of respiratory complications (see Table 3) and may require new strategies to improve their outcomes.

In conclusion, our data demonstrates that the number of days requiring supplemental O2 beyond 36 weeks PMA provides critical information about BPD severity that improves the prediction of respiratory morbidity after NICU discharge. We feel that incorporating O2 supplementation beyond 120 days into the definition and severity of BPD would be a useful clinical and research outcome. Nonetheless, it is important to emphasize that the total number of days requiring supplemental O2 is a clinical parameter that must be evaluated with extreme caution in prematurity. This clinical variable provides dual information about hypoxemia and the iatrogenic exposure to O2, a pathogenic factor in BPD^7,8^. The duration of supplemental O2 requirements may also indicate the presence of additional respiratory complications in premature infants. For instance, supplemental O2 may be part of the management of central breathing abnormalities^30,31^, or pulmonary vascular disease^29^, which are common and increasingly recognized complications of premature birth. Accordingly, we believe this important clinical variable must be defined and quantified objectively in each premature infant through a physiological challenge. More sophisticated algorithms for SpO2 analysis^33^, and the addition of other physiological parameters to understand ventilatory instability and cardio-respiratory coupling^30,31^, may further refine BPD severity assessment. Our studies provide compelling new evidence that these physiological tests to determine O2 requirements should be conducted at 36 weeks PMA and at later points (e.g. 90 days and 120 days) to improve BPD severity grading and risk stratification during and after NICU discharge.

## Supplementary information

Supplementary information
