# Supplementary material for: Validation of a new predictive model to improve risk stratification in bronchopulmonary dysplasia
Source: Sci Rep. 2020 Jan 17;10:613. doi: 10.1038/s41598-019-56355-5 (PMC6969113; doi:10.1038/s41598-019-56355-5)
Supplement: Supplementary file 1 — Supplementary information [file 41598_2019_56355_MOESM1_ESM.docx]

**Validation of a new predictive model to improve risk stratification in bronchopulmonary dysplasia**

Gustavo Nino, M.D., M.S.^a^, Awais Mansoor, Ph.D.^b^, Geovanny F. Perez, M.D.,M.S.^a^, Maria Arroyo,M.D.^a^, Xilei Xuchen, M.D.^a^, Jered Weinstock, M.D.^a^, Kyle Salka, M.S.^a^,^,^ Mariam Said, M.D ^c^, Ranniery Acuña-Cordero, M.D., M.S.^d^, Monica P. Sossa-Briceño, M.D., M.S.^e^, Carlos E. Rodríguez-Martínez M.D., M.S.^f,g^ and Marius Linguraru, Ph.D.^b^

**Study Subjects and Design**

**Primary cohort**

Our primary cohort was a study set to identify lung X-ray imaging and clinical predictors of respiratory hospitalizations in premature infants. All premature infants ≤32 weeks gestational age (GA) admitted to the NICU at Children’s National Health System (CNHS) in Washington, DC were considered eligible for this study.

We only included premature infants that had baseline CXRs taken routinely near discharge (between 36-41 weeks PMA). To quantify baseline BPD changes we selected CXRs without pneumothorax or pneumonia. However, all subjects were included independently of their history of pneumothorax or pneumonia in the NICU. All premature infants included had continued clinical care in CNHS for at least 12 months after discharge and had complete electronic medical record data (NICU and outpatient) to ascertain predictors of interest (e.g. days on supplemental O2) and main outcome (respiratory hospitalizations after discharge). We counted respiratory hospitalization as only those in which the primary complaint was any type of respiratory sign or symptom (e.g. cough, nasal/chest congestion, wheezing, respiratory distress, hypoxemia, etc). We excluded premature newborns with (1) congenital conditions that may affect lung development (such as congenital heart disease, congenital diaphragmatic hernia, cystic fibrosis, airway abnormalities); (2) immunodeficiency; (3) neuromuscular disorders; and (4) premature babies requiring tracheostomy and mechanical ventilation for long-term management of respiratory failure. Premature babies that required tracheostomy and mechanical ventilation were excluded because our primary cohort was a study set to identify lung X-ray imaging predictors of respiratory hospitalizations in premature infants and we were concerned that tracheostomy and mechanical ventilation could affect lung imaging analysis. This study was approved by the Institutional Review Board of Children’s National Medical Center, Washington, D.C.

**Validation cohort**

Our validation cohort was a study set to identify clinical predictors of respiratory hospitalizations in premature infants as part of Kangaroo Mother Care (KMC) program of the NICU of the Hospital Militar Central and the Hospital Universitario Clinica San Rafael. The study site was Bogota, the capital city of Colombia, a tropical LMIC located at an elevation of about 2650m (8660 ft) above sea level. The city's high altitude decreases atmospheric partial pressure of inspired oxygen, which subjects individuals to hypoxemia. The KMC program was developed to facilitate early discharge and long-term follow-up of premature patients, particularly for those requiring O2 beyond 36 weeks PMA. The KMC Program began in 1978 in Bogota, Colombia, in response to overcrowding and insufficient resources in NICU associated with high morbidity and mortality among low-birthweight infants. The intervention consists of continuous skin-to-skin contact between the mother and the infant, exclusive breastfeeding, and early home discharge in the kangaroo position.

We conducted a prospective cohort study that recruited premature infants (born at 36 weeks gestation or earlier) included in the KMC program. Parents of included infants had varied educational background and socioeconomic status, but all of them had at least 5 years of formal education, and some type of health insurance coverage. All premature infants in the KMC program were considered eligible for this study. Subjects were excluded if: 1) they expected to move out of Bogota within 2 years; 2) had incomplete data to ascertain predictors of interest (e.g. days on supplemental O2) and main outcome (respiratory hospitalizations after discharge); 3) did not have physiological room air challenges when completing 90 days and 120 days on supplemental O2; 4) failed to complete at least 4 visits during the first 12 months follow-up after NICU discharge. Scheduled ambulatory visits were conducted to monitor respiratory status, growth and perform physiological room air challenges to establish need for supplemental O2. The primary outcome was respiratory hospitalizations after NICU discharge and was defined exactly as in our primary cohort. The study protocol was approved by the local ethics board.

**Lung imaging analysis**

All individuals enrolled in our primary cohort had a baseline chest radiograph (CXR) performed near discharge (between 36-41 weeks PMA), which was used for lung disease quantification. The visual scoring was conducted blindly and independently by two pediatric pulmonologists (GN and GP) using a modified grading system described by Greenough et al. (12-14)

As shown in the table below, the CXR scoring system had three signatures of BPD: 1) fibrosis/interstitial opacities; 2) cystic/coarse elements; and 3) hyperinflation. We modified the initial score based on rib counting to determine hyperinflation to use diaphragm shape, which is also a surrogate of air-trapping. Based on the original Greenough’s score, each image was assigned a blinded total score (0-8) based on a global grading system for each of these lung disease parameters.

**Modified CXR score**

| **Score** | **0** | **1** | **2** | **3** | **4** |
| --- | --- | --- | --- | --- | --- |
| Fibrosis/interstitial opacities | 0 | 1 zone | 2 zones | 3 zones | 4 zones |
| Cystic elements | 0 | Small | Multiple and large |  |  |
| Hyperinflation: diaphragm shape | Normal curvature | Mild flattening | Complete flattening or concavity |  |  |

To facilitate the visual determination of lung textures by quadrants, we used two computer aided steps: 1) image intensity standardization algorithms; and 2) automatic lung segmentation. These algorithms were implemented prior to visual scoring using the ITK-Snap interactive software (<http://www.itksnap.org/>).

*Image Intensity Standardization*

Chest xrays collected using different scanners and protocol settings exhibit variable intensity ranges that pose a challenge for visual scoring. We used intensity standardization (mapping the acquired data to a predefined intensity profile) as a pre-processing step to alleviate this challenge. We implemented a pipeline based on a generic method for the intensity standardization of 2D/3D medical images with the multiscale curvelet transform. Briefly, during a training phase we decomposed the reference data into scale and orientation localized sub-bands using the multiscale curvelet transform, and calculated a reference energy value for each sub-band. Thereafter, the localized energy of each sub-band was iteratively scaled to the reference localized energy value from the training stage. We used this method in all images to standardize the intensity of the CXRs within the extracted lung field region.

*Automatic lung segmentation*

This step was particularly important for the determination of fibrosis/interstitial opacities, which is based on the number of zones (quadrants) affected. Automatic lung shape determination was also useful to enhance diaphragm contour for scoring of hyperinflation. The first step in our framework was the delineation of lung field from CXR. We used a weighted-shape partitioning approach by dividing the lung field into a set of partial shapes and learning a statistical shape model for each partition separately. Then, the lung field landmarks were divided into various consistent partitions using fuzzy c-means clustering. Next, we constructed a local appearance model consisting of three appearance features for every partition: (i) normalized intensity derivatives, (ii) three class fuzzy c-means, and (iii) elongated structure probability using the vesselness filter. We performed statistical shape model fitting for each partition and the optimal position for each landmark was determined by minimizing the Mahalanobis distance. We generated the final lung field segmentation by adding the different partitions and averaging the shape parameters of the overlapping landmarks. This method was used to have automated segmentation (quadrants) of 2D pediatric CXR images prior to visual scoring.

**Supplementary Table 1.**

Sensitivity, specificity, positive predictive value and negative predictive value for each data point of number of days on O2 in primary cohort (binary outcome respiratory hospitalization in the first year of life)

| Cutpoint | Sensitivity | Specificity | Classified | LR+ | LR- |
| --- | --- | --- | --- | --- | --- |
| ( >= 0 ) 100.00% 0.00% 43.62% 1.0000 | | | | | |
| ( >= 1 ) 97.56% 3.77% 44.68% 1.0139 0.6463 | | | | | |
| ( >= 2 ) 97.56% 4.72% 45.21% 1.0239 0.5171 | | | | | |
| ( >= 3 ) 97.56% 6.60% 46.28% 1.0446 0.3693 | | | | | |
| ( >= 4 ) 97.56% 7.55% 46.81% 1.0553 0.3232 | | | | | |
| ( >= 5 ) 97.56% 10.38% 48.40% 1.0886 0.2350 | | | | | |
| ( >= 6 ) 97.56% 11.32% 48.94% 1.1002 0.2154 | | | | | |
| ( >= 7 ) 96.34% 12.26% 48.94% 1.0981 0.2983 | | | | | |
| ( >= 8 ) 96.34% 17.92% 52.13% 1.1738 0.2041 | | | | | |
| ( >= 9 ) 96.34% 20.75% 53.72% 1.2157 0.1763 | | | | | |
| ( >= 10 ) 95.12% 20.75% 53.19% 1.2003 0.2350 | | | | | |
| ( >= 11 ) 95.12% 22.64% 54.26% 1.2296 0.2154 | | | | | |
| ( >= 12 ) 95.12% 24.53% 55.32% 1.2604 0.1989 | | | | | |
| ( >= 13 ) 95.12% 26.42% 56.38% 1.2927 0.1847 | | | | | |
| ( >= 15 ) 93.90% 26.42% 55.85% 1.2761 0.2308 | | | | | |
| ( >= 17 ) 93.90% 27.36% 56.38% 1.2927 0.2229 | | | | | |
| ( >= 19 ) 92.68% 27.36% 55.85% 1.2759 0.2675 | | | | | |
| ( >= 21 ) 92.68% 28.30% 56.38% 1.2927 0.2585 | | | | | |
| ( >= 22 ) 92.68% 29.25% 56.91% 1.3099 0.2502 | | | | | |
| ( >= 24 ) 91.46% 30.19% 56.91% 1.3102 0.2828 | | | | | |
| ( >= 26 ) 91.46% 31.13% 57.45% 1.3281 0.2742 | | | | | |
| ( >= 27 ) 91.46% 32.08% 57.98% 1.3465 0.2661 | | | | | |
| ( >= 30 ) 90.24% 33.02% 57.98% 1.3473 0.2955 | | | | | |
| ( >= 31 ) 87.80% 33.96% 57.45% 1.3296 0.3591 | | | | | |
| ( >= 32 ) 87.80% 34.91% 57.98% 1.3489 0.3494 | | | | | |
| ( >= 33 ) 87.80% 35.85% 58.51% 1.3687 0.3402 | | | | | |
| ( >= 34 ) 87.80% 38.68% 60.11% 1.4319 0.3153 | | | | | |
| ( >= 36 ) 86.59% 39.62% 60.11% 1.4341 0.3386 | | | | | |
| ( >= 37 ) 85.37% 39.62% 59.57% 1.4139 0.3693 | | | | | |
| ( >= 39 ) 84.15% 40.57% 59.57% 1.4158 0.3908 | | | | | |
| ( >= 40 ) 84.15% 42.45% 60.64% 1.4622 0.3734 | | | | | |
| ( >= 41 ) 84.15% 43.40% 61.17% 1.4866 0.3653 | | | | | |
| ( >= 42 ) 82.93% 43.40% 60.64% 1.4650 0.3934 | | | | | |
| ( >= 44 ) 81.71% 44.34% 60.64% 1.4680 0.4126 | | | | | |
| ( >= 46 ) 81.71% 46.23% 61.70% 1.5195 0.3957 | | | | | |
| ( >= 47 ) 80.49% 46.23% 61.17% 1.4968 0.4221 | | | | | |
| ( >= 48 ) 80.49% 47.17% 61.70% 1.5235 0.4137 | | | | | |
| ( >= 49 ) 79.27% 47.17% 61.17% 1.5004 0.4395 | | | | | |
| ( >= 53 ) 78.05% 48.11% 61.17% 1.5042 0.4562 | | | | | |
| ( >= 54 ) 78.05% 49.06% 61.70% 1.5321 0.4475 | | | | | |
| ( >= 55 ) 76.83% 49.06% 61.17% 1.5081 0.4723 | | | | | |
| ( >= 56 ) 75.61% 49.06% 60.64% 1.4842 0.4972 | | | | | |
| ( >= 60 ) 75.61% 50.00% 61.17% 1.5122 0.4878 | | | | | |
| ( >= 61 ) 74.39% 51.89% 61.70% 1.5462 0.4936 | | | | | |
| ( >= 62 ) 74.39% 52.83% 62.23% 1.5771 0.4848 | | | | | |
| ( >= 63 ) 74.39% 53.77% 62.77% 1.6093 0.4763 | | | | | |
| ( >= 65 ) 74.39% 54.72% 63.30% 1.6428 0.4680 | | | | | |
| ( >= 66 ) 74.39% 55.66% 63.83% 1.6777 0.4601 | | | | | |
| ( >= 67 ) 74.39% 57.55% 64.89% 1.7523 0.4450 | | | | | |
| ( >= 68 ) 74.39% 58.49% 65.43% 1.7921 0.4378 | | | | | |
| ( >= 69 ) 74.39% 59.43% 65.96% 1.8338 0.4309 | | | | | |
| ( >= 70 ) 74.39% 60.38% 66.49% 1.8775 0.4242 | | | | | |
| ( >= 71 ) 74.39% 61.32% 67.02% 1.9233 0.4176 | | | | | |
| ( >= 72 ) 73.17% 61.32% 66.49% 1.8917 0.4375 | | | | | |
| ( >= 74 ) 73.17% 64.15% 68.09% 2.0411 0.4182 | | | | | |
| ( >= 75 ) 71.95% 66.98% 69.15% 2.1791 0.4188 | | | | | |
| ( >= 78 ) 70.73% 67.92% 69.15% 2.2052 0.4309 | | | | | |
| ( >= 79 ) 69.51% 68.87% 69.15% 2.2328 0.4427 | | | | | |
| ( >= 80 ) 68.29% 70.75% 69.68% 2.3352 0.4481 | | | | | |
| ( >= 81 ) 64.63% 71.70% 68.62% 2.2837 0.4933 | | | | | |
| ( >= 82 ) 64.63% 72.64% 69.15% 2.3625 0.4869 | | | | | |
| ( >= 83 ) 63.41% 73.58% 69.15% 2.4007 0.4972 | | | | | |
| ( >= 84 ) 63.41% 74.53% 69.68% 2.4896 0.4909 | | | | | |
| ( >= 85 ) 62.20% 74.53% 69.15% 2.4417 0.5073 | | | | | |
| ( >= 89 ) 60.98% 75.47% 69.15% 2.4859 0.5171 | | | | | |
| ( >= 90 ) 60.98% 77.36% 70.21% 2.6931 0.5045 | | | | | |
| ( >= 91 ) 59.76% 78.30% 70.21% 2.7540 0.5140 | | | | | |
| ( >= 92 ) 59.76% 80.19% 71.28% 3.0163 0.5019 | | | | | |
| ( >= 93 ) 58.54% 80.19% 70.74% 2.9547 0.5171 | | | | | |
| ( >= 95 ) 56.10% 81.13% 70.21% 2.9732 0.5411 | | | | | |
| ( >= 97 ) 54.88% 82.08% 70.21% 3.0616 0.5498 | | | | | |
| ( >= 98 ) 54.88% 83.02% 70.74% 3.2317 0.5435 | | | | | |
| ( >= 99 ) 54.88% 83.96% 71.28% 3.4218 0.5374 | | | | | |
| ( >= 100 ) 53.66% 83.96% 70.74% 3.3458 0.5519 | | | | | |
| ( >= 102 ) 52.44% 83.96% 70.21% 3.2697 0.5665 | | | | | |
| ( >= 103 ) 51.22% 83.96% 69.68% 3.1937 0.5810 | | | | | |
| ( >= 106 ) 50.00% 83.96% 69.15% 3.1176 0.5955 | | | | | |
| ( >= 107 ) 47.56% 83.96% 68.09% 2.9656 0.6246 | | | | | |
| ( >= 108 ) 46.34% 84.91% 68.09% 3.0701 0.6320 | | | | | |
| ( >= 110 ) 43.90% 84.91% 67.02% 2.9085 0.6607 | | | | | |
| ( >= 112 ) 42.68% 85.85% 67.02% 3.0163 0.6676 | | | | | |
| ( >= 113 ) 41.46% 86.79% 67.02% 3.1394 0.6744 | | | | | |
| ( >= 114 ) 40.24% 86.79% 66.49% 3.0470 0.6885 | | | | | |
| ( >= 115 ) 40.24% 87.74% 67.02% 3.2814 0.6811 | | | | | |
| ( >= 116 ) 39.02% 87.74% 66.49% 3.1820 0.6950 | | | | | |
| ( >= 117 ) 36.59% 88.68% 65.96% 3.2317 0.7151 | | | | | |
| ( >= 118 ) 35.37% 89.62% 65.96% 3.4080 0.7212 | | | | | |
| ( >= 119 ) 34.15% 90.57% 65.96% 3.6195 0.7271 | | | | | |
| ( >= 120 ) 31.71% 91.51% 65.43% 3.7344 0.7463 | | | | | |
| ( >= 122 ) 28.05% 91.51% 63.83% 3.3035 0.7863 | | | | | |
| ( >= 124 ) 26.83% 91.51% 63.30% 3.1599 0.7996 | | | | | |
| ( >= 125 ) 26.83% 92.45% 63.83% 3.5549 0.7914 | | | | | |
| ( >= 126 ) 25.61% 92.45% 63.30% 3.3933 0.8046 | | | | | |
| ( >= 127 ) 25.61% 93.40% 63.83% 3.8780 0.7965 | | | | | |
| ( >= 128 ) 23.17% 93.40% 62.77% 3.5087 0.8226 | | | | | |
| ( >= 129 ) 21.95% 93.40% 62.23% 3.3240 0.8357 | | | | | |
| ( >= 131 ) 20.73% 93.40% 61.70% 3.1394 0.8487 | | | | | |
| ( >= 138 ) 20.73% 95.28% 62.77% 4.3951 0.8319 | | | | | |
| ( >= 142 ) 18.29% 95.28% 61.70% 3.8780 0.8575 | | | | | |
| ( >= 144 ) 17.07% 95.28% 61.17% 3.6195 0.8703 | | | | | |
| ( >= 147 ) 14.63% 95.28% 60.11% 3.1024 0.8959 | | | | | |
| ( >= 148 ) 10.98% 96.23% 59.04% 2.9085 0.9252 | | | | | |
| ( >= 150 ) 10.98% 97.17% 59.57% 3.8780 0.9162 | | | | | |
| ( >= 152 ) 9.76% 99.06% 60.11% 10.3414 0.9110 | | | | | |
| ( >= 157 ) 8.54% 99.06% 59.57% 9.0488 0.9233 | | | | | |
| ( >= 163 ) 7.32% 100.00% 59.57% 0.9268 | | | | | |
| ( >= 165 ) 6.10% 100.00% 59.04% 0.9390 | | | | | |
| ( >= 172 ) 4.88% 100.00% 58.51% 0.9512 | | | | | |
| ( >= 189 ) 3.66% 100.00% 57.98% 0.9634 | | | | | |
| ( >= 199 ) 2.44% 100.00% 57.45% 0.9756 | | | | | |
| ( >= 232 ) 1.22% 100.00% 56.91% 0.9878 | | | | | |
| ( > 232 ) 0.00% 100.00% 56.38% 1.0000 | | | | | |
